# Halophilic Plant-Associated Bacteria with Plant-Growth-Promoting Potential

**DOI:** 10.3390/microorganisms11122910

**Published:** 2023-12-02

**Authors:** McKay Meinzer, Niaz Ahmad, Brent L. Nielsen

**Affiliations:** 1 Department of Microbiology & Molecular Biology, Brigham Young University, Provo, UT 84602, USA; mckaymeinzer@gmail.com; 2 National Institute for Biotechnology and Genetic Engineering College (NIBGE-C), Pakistan Institute for Engineering and Applied Sciences (PIEAS), Faisalabad 38000, Pakistan; niazbloch@yahoo.com

**Keywords:** PGPB, salt tolerance, halophilic bacteria, *Halomonas*, *Kushneria*

## Abstract

The salinization of soils is a growing agricultural concern worldwide. Irrigation practices, drought, and climate change are leading to elevated salinity levels in many regions, resulting in reduced crop yields. However, there is potential for a solution in the microbiome of halophytes, which are naturally salt-tolerant plants. These plants harbor a salt-tolerant microbiome in their rhizosphere (around roots) and endosphere (within plant tissue). These bacteria may play a significant role in conferring salt tolerance to the host plants. This leads to the possibility of transferring these beneficial bacteria, known as salt-tolerant plant-growth-promoting bacteria (ST-PGPB), to salt-sensitive plants, enabling them to grow in salt-affected areas to improve crop productivity. In this review, the background of salt-tolerant microbiomes is discussed and their potential use as ST-PGPB inocula is explored. We focus on two Gram-negative bacterial genera, *Halomonas* and *Kushneria*, which are commonly found in highly saline environments. These genera have been found to be associated with some halophytes, suggesting their potential for facilitating ST-PGPB activity. The study of salt-tolerant microbiomes and their use as PGPB holds promise for addressing the challenges posed by soil salinity in the context of efforts to improve crop growth in salt-affected areas.

## 1. Introduction

The human population is growing at an alarming rate, and it is projected to reach 10 billion people by 2050, placing significant stress on food production [1]. Among the various factors that negatively impact crop yields, salinity plays a crucial role. Soil salinity tends to increase under certain conditions, for example, when water drainage is inadequate, when irrigation water is mixed with brackish water (a practice that farmers are compelled to adopt due to limited water supplies), when there is insufficient rainfall/precipitation, and when other related factors come into play [2]. There is a great need for new strategies to alleviate the effects of salinity on crop production [1,3].

## 2. Plants and the Negative Impact of Salt Stress

### 2.1. Increasing Salinity of Soils and the Detrimental Effects of Salt on Plants

Crop cultivation worldwide is facing an escalating challenge posed by soil salinization—the accumulation of salts in soil. The high levels of salt in the soil induce ionic stress in plants and disrupt cellular pathways due to the increased concentration of Na^+^ ions. Salinity increases in irrigated areas due to soluble salts carried in irrigation water that remain in the soil after evaporation and transpiration. Unless these salts are leached from the soil, they accumulate to levels that are inhibitory to plant growth and may lead to soils becoming sodic. High levels of sodium cause degradation of soil structure that affects water and root penetration [4]. The escalating problem of salinization poses a significant threat to global food security, with approximately 700,000 hectares of arable land being abandoned annually due to this issue [3,5,6]. As of 2022, salinity affects an estimated 1 billion hectares of arable land globally, with the economic cost of soil salinity estimated to be over USD 27 billion annually [7]. High salt concentrations in soil adversely affect crop growth and yield, making it a prominent constraint in modern agriculture. In the United States alone, soil salinity is responsible for a loss of USD 3.1 billion annually in crop production [8]. Salinity-induced crop yield reduction is a worldwide phenomenon, with countries like Australia experiencing losses of up to 50% in wheat yields due to salinity [9].

Salinization severely hampers agricultural productivity and is further compounded by the consequences of climate change. The rising temperatures and erratic weather patterns resulting from climate change exacerbate the salt buildup, rendering vast expanses of once-fertile land unsuitable for cultivation. Estimations by the Food and Agriculture Organization (FAO) indicate that around 20% of the world’s agricultural land is currently affected by salinization, and if the current trend persists, this figure is projected to skyrocket to a staggering 50% by the year 2050 [10].

As salinization continues to wreak havoc on agricultural landscapes, the repercussions for global food production and sustainability have already become visible in the form of acute food shortage crises. Salinization’s far-reaching effects on water resources and biodiversity further compound the ecological consequences. Most traditional crops are sensitive to salt, leading to decreased yield [4]. The severity of increasing soil salinity is likely to intensify with growing food demand and degradation of prime agricultural land. These factors leave farmers in many parts of the world with only poor-quality land and/or poor-quality water to produce crops for human consumption and to feed animals.

High salinity is one of the most severe abiotic factors globally, causing significant damage to plant growth and the plant life cycle [6]. As a considerable portion of arable land worldwide succumbs to salinization, the ability to grow sufficient crops to provide for the growing human population is gradually declining. A number of approaches including genetic engineering have been undertaken to improve tolerance of crop plants to salt stress [11,12,13]. However, the contributions of the microbial community for plant adaptations to adverse environmental conditions have been largely ignored. There is a growing body of evidence that the plant-associated microbial community, particularly those with halophytes, may play a key role in conferring plant stress tolerance in a habitat-specific manner.

### 2.2. Effect of Salt Stress on Field Crops

The detrimental effects of salinity on plant health are profound. High salt levels disrupt the osmotic balance within plant cells, leading to reduced water uptake and turgor pressure [3,5]. This results in impaired photosynthesis, nutrient uptake, and overall plant growth. Additionally, salinity stress induces the production of reactive oxygen species (ROS) in plants, causing oxidative damage to cellular structures [5,6]. Furthermore, excess sodium and chloride ions, which are toxic to plants, accumulate in plant tissues, disrupting vital metabolic processes [6,14]. These challenges highlight the urgent need to develop salt-tolerant crop varieties and adopt sustainable agricultural practices to mitigate the economic and ecological consequences of soil salinity.

Almost all field crops, being glycophytes, are highly sensitive to salt stress. Unlike halophytes, these plants are unable to withstand salt stress, and consequently, their growth is severely affected due to salt-induced toxicity, resulting in huge yield losses [14].

In the presence of about 300 mM NaCl or higher, glycophytes undergo a stress response that includes greatly reduced seed germination or sometimes failure, diminished root proliferation, reduced photosynthesis, and poor plant growth [6,14]. Excessive salt uptake results in a situation termed ‘salt poisoning’, which leads to nutrient imbalance, damage to membranes causing electrolyte leakage, and altered metabolisms. Photosynthesis is particularly vulnerable to salt stress and has been shown to correlate with the degree of salt stress [15]. Under salt stress, light use efficiency of the photosynthetic processes decreases, and energy losses increase due to sensitivity of several enzymes to Na^+^ ion toxicity in a dose-dependent manner [16]. For example, an exposure to salt stress of ~300 mM has shown to cause a 50–70% loss in protein function [2]. The accumulation of Na^+^ can lead to a multitude of issues within the plant, including imbalance in cellular homeostasis, oxidative stress, increased reactive oxygen species (ROS) secretion, nutrient deficiency, interference with K^+^ and Ca^+^ functions, retarded growth, and the eventual death of the cell [2,14,17]. ROS are a byproduct of photosynthesis and cellular respiration and can lead to DNA damage.

### 2.3. Halophytes and Their Ability to Tolerate High Salt

Salt stress is a significant challenge for plants, impacting their growth and overall productivity. To adapt to high salt concentrations, plants have evolved a diverse array of mechanisms and traits that work in tandem to ensure their survival. This interplay between various strategies is crucial for maintaining cellular integrity and function under saline conditions. Halophytes are defined as plants that can complete their entire life cycle in salt concentrations of 200 mM NaCl or greater [17,18]. Halophytes have undergone evolutionary adaptations, showcasing a wide range of morphological, anatomical, and physiological strategies to flourish in exceptionally saline environments. These specialized adaptations enable halophytes to thrive in various challenging habitats, such as inland saline zones, coastal areas, salt marshes, salt lakes, and mangroves. These plants have developed a repertoire of mechanisms to successfully adapt to high salt concentrations. This section delves into these remarkable adaptive strategies, providing a comprehensive understanding of how plants cope with the challenges posed by salinity stress.

Osmotic adjustment is one of the foremost and fundamental mechanisms allowing plants to mitigate the adverse effects of salt stress. This process involves the accumulation of compatible solutes like proline, glycine betaine, and soluble sugars. These solutes serve multiple purposes, including maintaining cellular turgor and water balance. Proline, for instance, acts as an osmoprotectant by stabilizing cellular structures and scavenging free radicals. Similarly, glycine betaine helps in ion homeostasis maintenance while protecting cellular membranes from damage [19,20]. In conjunction with osmotic adjustment, efficient ion transport and sequestration systems are pivotal for maintaining ion homeostasis under salt stress. For example, the SOS (Salt Overly Sensitive) pathway plays a central role in ion transport, comprising SOS1, SOS2, and SOS3. SOS1, located in the plasma membrane, expels excess sodium ions from the cell. SOS2, a protein kinase, phosphorylates SOS1 and activates its ion transport function in the presence of SOS3, a calcium sensor protein. Furthermore, vacuolar ion sequestration involving NHX antiporters plays a crucial role in reducing the sodium load in the cytoplasm [21,22].

As salt stress leads to the accumulation of reactive oxygen species (ROS), it is imperative for plants to employ mechanisms for ROS scavenging. This is achieved through a complex network of antioxidant enzymes, including superoxide dismutase, catalase, and peroxidases. These enzymes work together to scavenge ROS, thereby preventing oxidative damage to plant cells [23]. Furthermore, non-enzymatic antioxidants such as ascorbate and glutathione play a crucial role in ROS detoxification [24,25].

Root morphology also significantly contributes to salt tolerance in plants. A recent study showed that salt tolerance in *Brassica juncea* was linked with reduction in root diameter, which, in turn, promotes root length, surface area, and root volume [26]. Halophytes, which thrive in saline environments, possess specialized root structures that actively exclude salt from entering the plant. These adaptations may include salt glands, aerenchyma, or the formation of root apoplastic barriers. By employing these strategies, plants effectively reduce the uptake of toxic ions, enhancing their salt tolerance [27].

In addition to these physiological adaptations, hormonal regulation plays a significant role in the plant’s response to salt stress. Plant hormones like abscisic acid (ABA) regulate stomatal closure, reducing transpiration and water loss under salt stress [28]. ABA also induces the expression of stress-responsive genes, thus triggering a cascade of adaptive responses. Understanding how halophytes survive and thrive in saline soils can be beneficial in increasing the amount of land that is available for cultivation. Halophytic cells typically contain more than 500 mM NaCl. Extreme halophytes such as *Tecticornia* contain NaCl concentrations as high as 2000 mM [6,29,30].

There are different categories of halophytes, and their mechanism for dealing with high salt concentrations varies drastically. The first type of halophytes excrete salt. These plants have special glandular cells that excrete the excessive salt out of the plant body. The second type of halophytes comprises succulents. Succulents use salt bladders, typically located on the leaf surface, that hold a large amount of water to counteract the osmotic potential of the salt. The last category of halophytes comprises obligate halophytes or true halophytes. These plants need salt in order to complete their life cycle, as they deal with the high salt concentrations by compartmentalizing different ions in cells and the whole plant [31]. With soil salinity reaching a concentration of 300–400 mM on average, the exploitation of halophytes and the genes they possess could help farmers feed the growing population with a dwindling supply of arable land. The ability of halophytes to deal with high salt concentrations is dependent on controlled uptake and compartmentalization of Na^+^, K^+^, and Cl^−^ [5,32]. The ability of halophytes to tolerate multiple stresses is of intense interest in rehabilitating soils for cultivation [5]. Like all plants, halophytes have an associated microbiome and, in the past 10–15 years, there has been growing interest in microbiomes associated with native halophytes and their potential for enhancing plant growth [33,34,35].

## 3. Bacteria and Their Association with Plants

### 3.1. The Relationship between Bacteria and Plants in the Rhizosphere

The rhizosphere is the area in soil that immediately surrounds the roots of plants [36,37]. The rhizosphere contains countless species and a vast diversity of microorganisms [36,38]. One important component of the rhizosphere is plant mucilage. Mucilage is excreted by plant root tissue and can serve as a carbon source for microorganisms. The amount and composition of the mucilage can have a large impact on the bacterial species that live in the rhizosphere [37,39,40]. By excreting mucilage, plants attract beneficial microbes to the rhizosphere and benefit from microbial ability to break down sugars, fix nitrogen, suppress pathogens, etc. Mucilage can also play a role in attracting and supplying sugars for halophilic bacteria. These halophilic rhizobacteria could then, in turn, aid plants in saline soils. Bacteria found to be associated with the roots and that stimulate plant growth are termed plant-growth-promoting rhizobacteria (PGPR), and along with those that become established within plant tissue as endophytes are more broadly termed PGPB (plant-growth-promoting bacteria).

**Figure 1:**
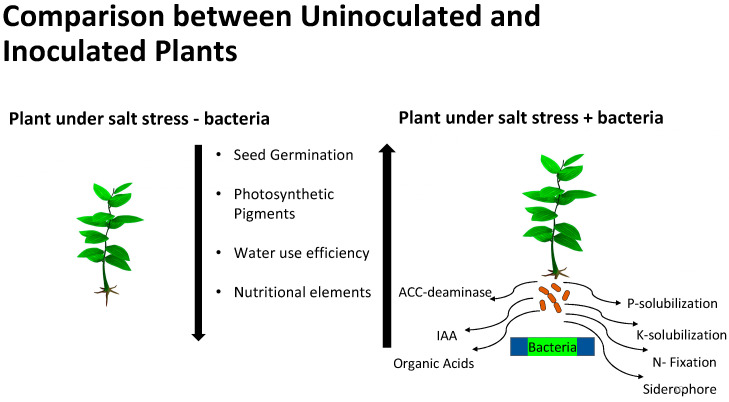
Effects of salt stress on plants and potential mechanisms of PGPR effects on plant growth in salty soils.

Species of bacteria and fungi that live within plant tissues without causing harm to the plant are defined as bacterial and fungal endophytes, respectively. There has been increasing interest in the role of endophytes in plant adaptation to different adverse environmental conditions in a variety of ways, as outlined in Figure 1 [41,42]. It has been well-established that microorganisms associated with plants thriving under harsh environmental niches play a crucial role in their adaptations to these suboptimal conditions [43]. The key mechanisms involved in such microbial-mediated adaptations to different stresses include modulation of phytohormones biosynthesis (auxins, cytokinins, gibberellins, abscisic acid, ACC deaminase (1-aminocyclopropane-1-carboxylate deaminase), brassinosteroids, and ethylene), accumulation of osmoprotectants (betaines, proline, and soluble sugars), upregulation of different defense genes, and production of secondary metabolites [43,44].

Plant hormones are chemical messengers that regulate various physiological processes in plants, including plant growth, development, and response to environmental stimuli such as light, temperature, and stress [45,46]. These hormones are master regulators, enabling plants to adapt and thrive in changing environmental conditions [47,48]. The diversity of phytohormones mirrors the multifaceted challenges that plants encounter in their environments. Each type of hormone is specialized in governing specific physiological responses, yet their interplay creates a sophisticated web of regulations that facilitate seamless coordination between different cellular and developmental processes. Moreover, plants often exhibit pleiotropy, where a single phytohormone can have widespread effects, influencing various aspects of plant life, while multiple hormones collaborate to finetune a particular biological event. This intricately woven system of phytohormonal control empowers plants to maintain homeostasis and optimize their growth in response to internal and external cues. In response to environmental changes, such as light, temperature, water availability, or the presence of pests and pathogens, plants dynamically adjust their phytohormone profiles. Studies have shown that in the presence of high concentrations of salt, production of auxin is severely limited. Plants grown in salty soils had insufficient auxin levels and suffered from stunted growth [48]. Different bacterial strains have been shown to induce hormone production and supplement those produced by the plant through the function of microRNAs (miRNAs) [47,49]. If a plant has insufficient hormone levels, the growth and development of the plant, as well as the fruit, can suffer and yield can be significantly decreased.

Indole-3-acetic acid (IAA) is a plant hormone that regulates the production of new root and shoot tissues. It has been shown that IAA produced by bacteria can induce adventitious shoot growth [44,47,50,51,52]. High salinity induces the utilization of 1-aminocyclopropane-1-carboxylic acid (ACC). ACC is a precursor for ethylene, a plant hormone that mediates a wide range of essential plant responses. However, at elevated levels, ethylene has a deleterious effect on root and shoot elongation, leaf expansion, and overall plant health [52]. ACC deaminase is an enzyme that breaks down ACC, preventing the production of ethylene, and it can alleviate the stress response in plants. ACC deaminase-producing bacteria can help aid the plant when it is under salt stress, and even help promote plant growth and antioxidant production [52], as has been shown for the French bean [53].

Microorganisms are known to produce over 20,000 different secondary metabolites [54]. These metabolites can affect the survival and performance of other organisms. Not only do endophytes produce secondary metabolites beneficial to plant growth and disease response but endophytes also produce novel biomolecules and plant growth promotors [55]. Because of these beneficial effects, the utilization of these endophytes holds potential to improve plant growth, particularly under adverse environmental conditions such as on marginal lands, and it may confer resilience to climate change.

### 3.2. Halophilic Bacteria with PGPB Potential

There are a considerable number of *Bacillus* strains that have been identified as having general PGPB activity (reviewed in [56]). To date, there has been some limited analysis of possible functions as ST-PGPB for some, which is briefly summarized here. One recent report suggests that a soil *Bacillus* strain is recruited to coastal halophytes by exudates from plant roots, but this work does not include plant growth stimulation studies [57]. There is also an interesting recent publication on combinations of a *Bacillus* strain with other bacteria including a *Pseudomonas* strain to promote plant growth [58]. Other work has shown the promise of a combination of bacterial and mycorrhizal fungal applications in enhancing soil fertility and rice production due to enhanced mineral uptake from the soil [59].

The microbial community found to be associated with halophytes growing in saline soils represents a rich source of ST-PGPB (salt-tolerant, halophilic, rhizobacteria, or endophytes) [60,61,62,63,64,65,66,67,68,69,70,71,72,73,74]. Improvement of soil health and potential in bioremediation have also been reported [48,61]. A combination of strains may provide synergistic benefits. For example, one recent study showed the value of using a consortium of multiple halophilic strains isolated from halophytes to improve the growth of several crops under saline conditions [75]. Other studies have shown beneficial effects of ST-PGPB in reducing salt stress and improving yield in rice [76,77]. PGPB strains that have ACC deaminase and ROS scavenging activity have been shown to contribute to amelioration of salinity stress [78]. These reports provide a strong foundation for the use of halophiles isolated from the rhizosphere or roots of halophytes as inocula to stimulate the growth of salt-sensitive crops [79,80]. Recently, an Actinomycete was found to have antifungal activity and alleviate salt stress in tomato [81]. Another Actinomycete was reported to promote the growth of a halophyte using irrigation with seawater. Some biofilm-forming strains of *Bacillus* have been shown to enhance the growth of maize in saline conditions [82,83]. Another example is *Bacillus subtilis* (GB03), which was identified as a rhizosphere bacterium that stimulates growth of white clover under saline conditions [84], and which has since been renamed *Bacillus amyloliquifaciens* GB03 [85,86]. This strain produces volatile compounds that enhance plant photosynthetic capacity and chlorophyll content and induces an elevation of endogenous sugar content and suppression of abscisic acid (ABA)-induced RNA transcripts. Some of these strains also aid in dealing with other types of abiotic stress [87]. *B. amyloliquifaciences* GB03 has been shown to stimulate tall fescue growth under nitrogen limitation by altering regulation of phytohormones and nutrient homeostasis [88,89]. Other effects include alterations of plant gene expression, including upregulated expression of the HKT1 sodium transporter gene in shoots and downregulated expression in roots, which results in lower sodium accumulation throughout the plant [84,90].

In addition to PGPR isolated from soil around plant roots, endophytes (bacteria growing within plant tissues) have also been identified that stimulate plant growth [80,91,92]. Mechanisms by which endophytes enhance plant growth are thought to include enhanced nutrient acquisition and changes in host plant gene expression. For example, ACC (1-aminocyclopropane-1-carboxylase) deaminase is a bacterial enzyme found in many endophytes that stimulates nutrient acquisition and plant growth by reducing the amount of ACC converted to ethylene, a known inhibitor of plant growth, in response to salt, drought, and other environmental stresses [80,93,94]. *Burkholderia phytofirmans* is an endophyte that alters plant gene expression to enhance growth of six of the eight cultivars of switchgrass that were tested [91]. Inoculation with this strain was found to induce wide-spread changes in gene expression in the plant host, including altered expression of some transcription factors that are known to regulate the expression of plant stress factor genes [95]. Other bacterial endophytes (species of *Sphingomonas*, *Pantoea*, *Bacillus*, and *Enterobacter*) have been identified that enhance the salt tolerance of hybrid elephant grass [69], likely because of enhanced nutrient acquisition and/or gene expression changes [96].

### 3.3. Bacterial Strategies to Overcome Salinity Stress

Salt stress is one of the largest abiotic factors that can impact growth of an organism, including bacteria, which are also susceptible to osmotic stress. Halophilic bacteria have multiple mechanisms to counteract the osmotic stress of saline environments. There are two main types of adaptation mechanisms that halophiles use to prevent desiccation in the presence of salt: accumulation of water-soluble organic compounds in the cytoplasm and controlling the flux of inorganic ions. The main way that bacteria control the flux of inorganic ions is by exporting K^+^ ions to offset the influx of Na^2+^ ions. In addition, many halophiles utilize accumulation of water-soluble organic compounds (ectoine, hydroxyectoine, betaine, and choline) to offset the osmotic stress of highly saline environments. The accumulation of these compounds, or osmolytes, helps to draw water into the bacterial cell, preventing desiccation of the cell [97]. Another strategy that is employed by a wide variety of halophiles is controlling the flux of inorganic ions in the cell. If a cell has an influx of inorganic ions, this can lead to desiccation of the cell and eventual death of the organism. One method to prevent this influx of ions, typically sodium ions, is to actively pump intracellular potassium outside of the cell. This potassium typically comes in the form of KCl, and the export of KCl helps to offset the influx of NaCl from the saline environment [97].

It appears that in addition to the mechanisms used by PGPBs in general, there may be different mechanisms for PGPB stimulation of plant growth in saline conditions depending on the plant and bacterial species involved. The mechanism(s) by which halophilic bacteria stimulate plant growth may involve production of volatile compounds or other signals that stimulate expression of genes to enhance growth via increased photosynthesis [98] and other processes, including increased expression of plant membrane ion transport proteins. Other mechanisms that have been proposed include, but may not be limited to, the following ([67], Figure 1 and Table 1):

- Some microbes produce biofilm/exopolysaccharides in the rhizosphere that trap water and nutrients and decrease plant uptake of sodium ions from the soil.
- Some microbes inhibit growth of fungi and plant pathogens and/or select a certain microbial community in the rhizosphere.
- Enhance plant access to nutrients.
- Some microbes function as phytostimulators to produce ABA, IAA, and other plant hormones that stimulate shoot formation and plant growth by enhancing expression of specific plant genes.
- Microbes can function as biofertilizers to produce nutrients or improve nitrogen fixation for the plant and/or enhance photosynthesis.
- Solubilization and translocation of ions such as phosphate and iron (by siderophores) to the plant. Phosphate and iron are critical to the plant but are often in low abundance or present only in inorganic forms that must be solubilized for use by plants ([82,83], Table 1).

### 3.4. Salt Tolerant Bacterial Genus Kushneria

While much of the published work has focused on *Bacillus* species or other Gram-positive bacteria, some Gram-negative bacteria also have been shown to have PGPB potential. Two that have been shown to stimulate plant growth of salt-sensitive plants in salty soil are *Kushneria* and *Halomonas* (Table 1). *Kushneria* is a genus of bacteria in the *Halomonadaceae* family and comprises halophiles. The genus *Kushneria* was formed in 2009 when *Halomonas marisflavi* along with two other *Halomonas* strains were moved into this novel genus [116]. One of the most notable features of *Kushneria* is its ability to grow in high salt concentrations. Studies have shown that some species of *Kushneria* can grow in up to 25% NaCl concentration, which is higher than the salt concentration of seawater. This makes the genus an interesting subject of research for its potential use in bioremediation of saline soils, as well as in the production of salt-tolerant crops. Strains of *Kushneria* have been isolated from a variety of different salty environments, including a solar saltern, the leaves of black mangroves, sea water, salt mines, cured meats, and salt fermented foods [117,118,119,120,121,122,123]. Many species in this genus are adapted to hypersaline environments, and different strains have been isolated from the rhizosphere as well as the endosphere of halophytes [119,124]. These bacteria exhibit the ability to produce a variety of osmolytes, bioactive compounds (including betaine and ectoine that help protect from stress), and plant growth hormones [119,124]. Certain *Kushneria* species have been found to promote plant growth and act as biofertilizers or may function in phytoremediation, especially in saline agricultural soils [125]. A study by Parida and Das [126] revealed that *Kushneria* sp. NRCC 31,399 enhanced the growth of rice plants under saline conditions, indicating its potential use as a bioinoculant for salt-affected agricultural lands. *Kushneria* species are known for their ability to accumulate compatible solutes, which are small organic molecules that help the bacteria to cope with osmotic stress. These solutes have potential applications in various industries. In a study by Kulkarni et al. [127], *Kushneria* sp. GSB1 was found to produce the compatible solutes ectoine and hydroxyectoine in considerable amounts, highlighting the biotechnological potential of this genus in the production of these valuable compounds. Studies have shown that some species of *Kushneria* produce compounds that exhibit antimicrobial activity against various pathogenic bacteria and fungi. This suggests that *Kushneria* may have potential applications in the development of novel antibiotics and antifungal agents. *Kushneria* strains have been isolated from both the endosphere and the rhizosphere of plants and many exhibit the ability to produce a variety of plant hormones (Table 1). These examples demonstrate the diverse and promising biotechnological applications of *Kushneria* species. Continued research and exploration of this salt-tolerant bacterial genus may uncover even more practical uses in the future.

*Kushneria* and *Halomonas* bacteria have been isolated from a halophyte, Salicornia, in Tunisia, and some have been shown to have plant growth promotion activity [63]. A *Kushneria marisflavi* isolate in combination with *Pseudomonas stutzeri* was found to reduce salinity-stress-induced damage in lettuce and barley [101]. Despite the potential applications of *Kushneria*, much of its biology remains poorly understood. Further research is needed to fully elucidate the metabolic capabilities and genetic makeup of the genus. This would not only provide insights into the biology of *Kushneria* but also pave the way for the development of new biotechnological applications of the genus.

### 3.5. Salt-Tolerant Bacterial Genus Halomonas

*Halomonas* bacteria are able to grow in high-salt conditions and at high pH values. *Halomonas* can also resist contamination by other microbes, due to its ability to grow in highly saline and alkali conditions [91]. *Halomonas* spp. have been isolated from the endosphere of different plants, shrubs, and trees. They have been identified as Gram-negative, aerobic with yellow pigmentation, and are rod shaped [128]. Strains of *Halomonas* have been isolated from a variety of highly saline environments including salt marshes, the endosphere of halophytes, salt-cured meats, and fermented foods [125,128,129,130,131]. Bacteria from this genus have been found that produce a wide variety of diverse biochemicals and exopolysaccharides (EPSs) [129,130]. Table 1 includes other *Halomonas* strains that have been reported.

Some *Halomonas* species isolated from plants have been shown to have potential as PGPB [132,133] or in phytoremediation to improve soils [125] (Table 1). A collection of halotolerant microbes isolated from *Salicornia ramiosissima* [132] contains some isolates with PGPB activity. Two *Halomonas* and two *Bacillus* strains were identified from the rhizosphere of quinoa that have different activities as PGPB, and it was shown that these strains exhibit beneficial traits that are salt-regulated [133].

A consortium of five *Halomonas* strains was shown to improve salt tolerance of rice [106], suggesting that combinations of two or more strains may provide synergism. In wheat, inoculation with *Halomonas* sp. 3H led to an increase in chlorophylls, carotenoids, sugars, and phenolics [108]. *Halomonas* sp. MAN5 improved root growth of *Sesuvium portulcastrum* [110]. In separate studies, several other *Halomonas* strains have been shown to enhance salt tolerance in purple basil [111], maize [112], chickpea [131], and sunflower [114]. One *Halomonas* strain, *H. ventosae* JPT10, was tested with multiple plants and enhanced salt tolerance in foxtail millet, soybean, tomato, wheat, and maize [115], indicating that some strains may serve as PGPB for multiple crop species. In earlier work, our research group isolated many halophilic bacteria from halophytes near Utah Lake in Utah, including Halomonas and Kushneria strains, some of which were found to have ST-PGPB activity [102].

## 4. Conclusions

Salt stress is the single largest abiotic factor that determines the growth and production of plants. Worldwide, arable lands are becoming increasingly saline and with the increase in salinity, crop production is decreasing and many farmers are struggling to manage these conditions. Recently, researchers have been interested in determining how halophilic bacteria can aid plants in saline soils. Two halophilic genera of interest are *Kushneria* and *Halomonas.* These bacteria have been isolated from a variety of saline environments, including the plant tissues and associated rhizosphere of halophytes. A variety of species within both genera have been shown to produce plant hormones and osmoprotectants. The application of halophiles as plant-growth-promoting bacteria (PGPB) has shown much promise, but much more needs to be done for successful use in the field. *Kushneria* and *Halomonas* have been tested in a small number of plant growth stimulation research studies to date, and these genera have the potential to make a significant impact on growth promotion of crops in saline soils.

## Figures and Tables

**Table 1:** List of selected studies reporting improved salt tolerance of field crops or phytoremediation by *Kushneria* and *Halomonas* species.

Bacterial Species	Applications	Source of the Strain(s)	Experimental Conditions/Formulations/Outcome	Reference
*Kushneria* Species				
*Kushneria* sp. M3	Bioremediation of saline soil polluted with petroleum hydrocarbons	The bacteria were isolated from the salt water and sediment of Red Sea, Jeddah, Saudi Arabia.	Bacterial consortium in continuous stirred tank reactor with petroleum refinery wastewater under saline condition (40 g/L NaCl concentration). Almost complete and 90% degradation of low- and high-molecular-weight PAHs, respectively, was observed.	[99]
*Kushneria* sp. *YCWA18*	Increased germination under NaCl-alkali conditions as well as growth of *Suaeda Salsa* plants	The bacterium was isolated from the sediment of Daqiao saltern on the eastern coast of China.	The plants were inoculated with the bacteria and grown under NaCl-alkali regimes. A P solubilization of 616.98 mg/L was observed after 48 h. The germination was also improved. Bacteria did not show any effect under low salt-alkali conditions.	[100]
*Kushneria marisflavi*	Lowering salt-induced toxicity in barley, lettuce, and sunflower	Information about identification of bacterial strains not mentioned.	The seeds of the plants were inoculated with the bacterial culture, which was prepared using cells suspended in 2% NaCl solution, keeping an OD_600_ = 0.5 at 25 °C. The plants were grown in pots filled with sterilized sand and irrigated with NaCl-supplemented Hoagland’s solution. Plants were inoculated with bacterial culture twice. In the first treatment, seeds were soaked in bacterial culture for 45 min while the second inoculum was given after two weeks by adding 1 mL of bacterial culture to the medium.	[101]
*Kushneria marisflavi*	Improved alfalfa growth under salinity	Isolated from the soil and roots of *Salicornia rubra*, *Sarcocornia utahensis*, and *Allenrolfea occidentalis.*	Alfalfa seedlings grown at 1% NaCl concentration were inoculated with strains. The bacterial treatment of seedlings stimulated root growth in alfalfa up to 2.6-fold and a 21% increase in fresh weight compared to untreated controls.	[102]
*Kushneria* sp.	Elevated salt stress in rice	Different Bacteroidota and Actinobacteriota strains from *Avicennia marina* phyllosphere and rhizosphere were isolated.	The *Kushneria* strains inoculated were able to promote the growth of rice seedlings (root length, shoot length, and plant length) under 100 mM NaCl conditions by dissolving organic phosphorus and fixing nitrogen. The salt stress was applied by treating rice seeds with or without 100 mL NaCl solution while the inoculum was applied by adding 10 mL bacterial solution to the plates.	[103]
*Kushneria*	Enhanced salt tolerance in Chia	Different bacterial strains were isolated from the rhizosphere of *Adesmia horrida* (Fabaceae), *Senecio punae* (Asteraceae), and *Pappostipa frigida* (Poaceae).	Chia seeds were grown on half-strength MS medium supplemented with or without 50 and 100 mM NaCl. The bacterial strains were inoculated by adding a 20 µL bacterial culture to each plate, which were also prepared in half-strength MS medium, 0.2% sucrose, and 0.8% agar by culturing at 30 °C.	[104]
*Kushneria BSSM27*	Alleviation of salt stress in durum wheat	The strains were isolated from the rhizosphere and roots of *Halocnemum strobilaceum*.	The cultures were prepared on YESA (Yeast Extract Sucrose Agar) medium with 2% sucrose and incubated at 30 °C. Plants were grown in pots, and inoculum (10 mL, OD_600_ = 0.6–0.8) was applied after coleoptile emergence. The salt stress was applied by irrigating plants with or without 100 mM and 200 mM NaCl solution every other day for 21 days.	[105]
***Halomonas* species**				
*Halomonas* sp. Exo1	Improved salt tolerance of rice plants in saline soils	The rhizobacteria was isolated from *Avicennia marina* rhizosphere of Indian Sundarbans.	The bacterium was applied either alone or in consortium with five other *Halomonas* strains. The treatment was applied twice: one before sowing to the seeds, and a second one at the time of transplantation of the seedlings into pots. Plants were grown in soil containing either 0.1% (*w*/*w*) NaCl or 0.2% (*w*/*w*) NaCl in the presence of arsenic. The treatment of bacterium alone did not yield any noticeable effect on germination of plants. A slight increase, however, occurred in the presence of arsenic.	[106]
*Halomonas campaniensis 3H*	Removal of nitrous oxide	The strain was isolated from a eutrophic saline lake sediment.	For denitrification experiments, the strain 3H was cultured in a minimal medium supplemented with 50 g/L NaCl, ammonia, nitrate, or nitrite as the sole nitrogen source, with constant shaking at 150 rpm and 30 °C. Cells from the logarithmic phase were diluted to OD_600_ = 0.02. Uninoculated medium was used as a control. An incubation of 96 h resulted in complete removal of ammonia. Further, addition of Cu^+2^ stimulated growth of 3H cells.	[107]
*Halomonas* sp. *3H*	Salt tolerance in wheat	The strains were isolated from salt-tolerant rhizosphere.	A pot experiment was performed to assess the growth-promoting potential of the identified strains. Bacterial cultures were applied to wheat by soaking wheat seeds in bacterial cultures for 3–4 h. Plants without bacterial inoculation were used as control. *Halomonas* cell treatment significantly increased chlorophylls, carotenoids, and soluble sugars. Likewise, a beneficial effect on phenolic contents was also observed.	[108]
*Halomonas* sp. *B01*	Removal of nitrogen (N) from high-salinity wastewaters	The strains were isolated from a saltern pool in Dalian, China.	The cells were cultured in a medium containing 30, 60, 90, and 120 g/L NaCl. For nitrogen removal, the strain was cultured in 5 mL media at 30 °C, and 1% of the cultures were inoculated in 300 mL flasks containing 30 mL of N removal medium; the SND was performed at 30 °C in a rotary shaker at 90 rpm. The strain was able to remove nitrogen in a time-dependent manner as well as a NaCl-dose-dependent manner. The removal efficiency of the strain was higher at high NaCl compared to lower concentrations, reaching as high as 90% in 96 h.	[109]
*Halomonas* sp. *MAN5*	Enhanced root growth of *Sesuvium portulacastrum* under saline and heavy metal stress	The strains were isolated from soil samples collected from mangrove rhizosphere.	A pot experiment was conducted to study improvement in salt tolerance of *S. portulacastrum*. A 10 mL bacterial culture was used. Plants were irrigated with 2% NaCl saline water. The treatment was continued for 1 month and a number of plant growth parameters were recorded. The root growth and dry weights of the plants were increased 4-fold and 5-fold, respectively.	[110]
*Halomonas* sp.	Salt tolerance in purple basil	No information on how the strains were isolated was provided.	Bacterial cultures were prepared by adding 7% NaCl to LB broth. Basil plants were grown in pots filled with 0.5 L perlite. Bacterial solutions were applied after the emergence of cotyledons while salinity treatments were initiated after plants reached the 6–8 leaf stage. A one-fourth strength Hoagland’s solution containing 0, 50, 100, or 150 mM NaCl was used for irrigation. The stress was applied for three weeks. Different plant growth attributes were monitored. *Halomonas* treatment alone had no significant effect on plant growth. However, when used in combination with *Azobacter* sp., it significantly improved the growth parameters under even at 150 mM NaCl stress applied.	[111]
*Halomonas* sp.	Salt tolerance in maize	No information regarding the isolation of bacterial strains was provided.	The bacterial cultures for inoculation were prepared by culturing in LB medium containing 0.5 M NaCl at 37 °C overnight. To study the effect of salt tolerance in maize, plants were grown in pots and irrigated with 0, 50, 100, and 200 mM salt solution. Seeds were sterilized and inoculated with 10 mL bacterial suspension for 30 min. Uninoculated seeds were used as control. Seedlings were harvested after 15 days and different growth parameters were recorded. Bacterial treatments significantly improved growth of treated plants under NaCl stress. For example, an increase of up to 210% was noted in germination, an up to 40% increase in shoot length, and an up to 137% increase in root length compared to untreated controls.	[112]
*Halomonas variabilis (HT1)*	Improved growth of chickpea under salinity	No information provided about the isolation of strain.	The bacterial cells were applied by incubating chickpea seeds for 30 min. Plants were grown in pots containing 0, 50, 100, and 200 mM NaCl per gram of soil. Seedlings were harvested after 15 days, and several parameters including seedling length (cm), fresh weight (mg per seedling), and dry weight (mg per seedling) were noted. Bacterial inoculation stimulated germination by 152%. A 50% increase was noted in germination rate. The bacterial strain also positively increased both the fresh weight and dry weight by 153% and 1988% compared to control under salt stress. Soluble sugar contents were increased by 46% and protein contents were increased 107% under salt stress. A notable feature observed during the study was the soil aggregation to plant roots under salt stress. The *Halomonas* strain increased 666% in soil aggregation under NaCl stress.	[113]
*Halomonas (MK873884)*	Enhanced growth of alfalfa under salinity	Cells isolated from rhizosphere of halophytic species *Salicornia rubra*, *Sarcocornia utahensis*, and *Allenrolfea occidentalis*.	Alfalfa seedlings were first germinated in sterile water, and seedlings were shifted to magenta boxes containing autoclaved soil. Then, 100 mL half-strength Hoagland’s solution supplemented with 1% NaCl and 1 mL of bacterial suspension was added to each box. A second experiment was carried out in pots in a greenhouse. This time, 1 mL of PBS buffer with or without 1 mL of bacterial culture was added to each pot. Salt stress was initiated after 1 week of bacterial inoculation. For salt stress, plants were irrigated with or without 1% NaCl solution The stress was applied for one month. After one month, plants were harvested and different growth attributes noted. Significant changes in growth under salt stress were observed in plants treated with bacterial cells compared to untreated controls under similar conditions. For example, an up to 21% increase in fresh weight and up to a 2.6-fold increase in root length were observed in treated plants under stress conditions.	[102]
*Halomonas BSSM328*	Alleviation of salt stress in durum wheat	The strains were isolated from the rhizosphere and roots of *Halocnemum strobilaceum*.	The cultures were prepared on YESA (Yeast Extract Sucrose Agar) medium with 2% sucrose and incubated at 30 °C. Plants were grown in pots, and inoculum (10 mL, OD_600_ = 0.6–0.8) was applied after coleoptile emergence. The salt stress was applied by irrigating plants with or without 100 mM and 200 mM NaCl solution after every other day for 21 days.	[105]
*Halomonas venusta*	Enhanced plant growth in sunflower	No information provided about the isolation and purification of strains.	The *Helianthus annuus* seeds were treated with or without bacterial cells cultured at 37 °C. Both the treated and untreated seed were grown in pots and harvested after one month. Plants treated with bacterial cells showed significant improvement in different plant growth attributes, such as shoot length (+136%), leaf number (+52%), protein content (+57%), and flower diameter (+31.4%). Likewise, a positive and statistically significant effect was observed on chlorophyll concentration.	[114]
*Halomonas ventosae* JPT10	Promotes salt tolerance in foxtail millet, soybean, tomato, wheat, and maize	The cells were isolated from *Suaeda salsa* rhizosphere.	The cells were cultured in 15 mL LB medium containing 2 M NaCl at 28 °C for 24–48 h. The salt stress experiment was carried out in pots. The plants were irrigated with or without 100, 150, 200, and 250 mM NaCl solution. For bacterial inoculations, a 200 mL bacterial suspension was added to each pot. Bacterial treatment significantly improved plant growth under salt stress. The foxtail bacterial-treated plants accumulated fewer levels of OPDA, JA, MeJA, and ROS compared to untreated plants. Likewise, maize, wheat, soybean, and tomato bacterial-treated seedlings showed faster growth rates, and produced longer shoots and roots and higher fresh weights compared to untreated plants.	[115]

## Data Availability

See cited references for original data.
